# Microbial Pressure and Social Immunity: Bumble Bees Increase Brood Hygiene After Exposure to a *Bacillus thuringiensis* ‐Based Biopesticide

**DOI:** 10.1002/ece3.73844

**Published:** 2026-06-11

**Authors:** Michelle Scheffler, Karoline Wueppenhorst, Doreen Babin, Abdulrahim T. Alkassab, Silvio Erler

**Affiliations:** ^1^ Julius Kühn Institute Institute for Bee Protection Braunschweig Germany; ^2^ Technische Universität Braunschweig Zoological Institute Braunschweig Germany; ^3^ Julius Kühn Institute Institute for Epidemiology and Pathogen Diagnostics Braunschweig Germany

**Keywords:** *Bacillus thuringiensis*, *Bombus terrestris*, hygienic behaviour, larvae ejection, necrophoresis, social immunity

## Abstract

To inhibit the spread of potential pathogens and parasites, social insects exhibit hygienic behaviour, the removal of dead or affected brood. Studies on bumble bees performing this behaviour are limited, compared to honey bees, termites, and ants. With changes in agricultural practices, bees are exposed more often to bioinsecticides, especially microorganisms, which they bring into their nests and might cause damage to brood and adult bees. In a field feeding study, we explored whether an exposure to a biopesticide, comprising the bacterium *Bacillus thuringiensis* ssp. *aizawai* (strain: ABTS‐1857), affects colony development and hygienic behaviour of *Bombus terrestris* . Bumble bee colonies were studied in three groups: (1) repeated exposure (5 times) over 18 days to the plant protection product containing *B. t. a.* ABTS 1857, (2) artificial manipulation of the brood nest by wounding larvae to induce larvae removal, and (3) untreated control. Removed larvae were sampled daily and sorted into different weight and colour categories to analyse larval developmental stages. Over more than 3 weeks, we detected a much higher larvae removal in the *B. thuringiensis* treated colonies, which started 10 days after first exposure. Most of the removed larvae were light in weight (11–60 mg) and white coloured. Ejected larvae from the controls and brood manipulated colonies were all tested negative for *B. thuringiensis* , whereas biopesticide treated colonies were positive (PCR detection following selective plating). Regular colony assessments revealed no differences in colony development or production of new sexuals among all treatment groups, but heavier colonies removed more larvae. Further studies should focus on potential mechanisms, for instance the chemical ecology, behind the bioinsecticide induced hygienic behaviour.

## Graphical abstract

In social insect colonies, diseased or dead brood is identified and removed by workers to prevent the infection of the whole colony. Here, bumble bee larvae exposed to a bacterium ( *Bacillus thuringiensis* ssp. aizawai (strain: ABTS‐1857)) based biopesticide were removed more frequently than wounded or untreated control larvae. This shows that bumble bee colonies can regulate larvae removal behaviour to guarantee successful colony development and to reduce potential burden by sick individuals.

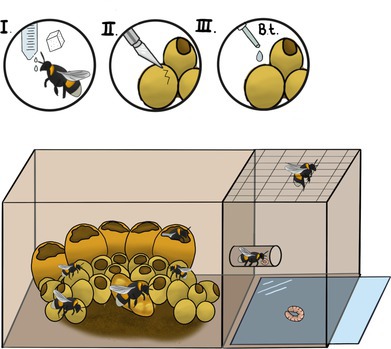

## 1 Introduction

Social insect colonies are characterised by group‐defence against parasites and predators, reproductive division of labour, cooperative brood care and highly efficient foraging (Wilson 1971). However, with many highly‐related individuals living in close proximity in a colony, parasites and pathogens can spread quickly and easily between different castes, sexes and developmental stages (Schmid‐Hempel 1995). To recognise and remove infected individuals, social insects have developed a collective immune defence system, so‐called social immunity (Cremer et al. 2007). Such a behavioural adaptation minimises the transmission of pathogens and parasites, and consequently disease outbreaks, in their colonies. The underlying mechanism is known as hygienic behaviour, and has effectively been demonstrated in honey bee brood for infections with the causative agent of chalkbrood disease (*Ascosphaera* spp., Swanson et al. 2009), American foulbrood ( *Paenibacillus larvae* , Rothenbuhler 1964), European foulbrood ( *Melissococcus plutonius* , Bailey 1960), and infestations with the ectoparasitic mite *Varroa destructor* (Mondet et al. 2016). Related behaviour, like removing dead individuals from the nest, guarding the nest entrance or foraging for antimicrobial substances has been well studied in ants, termites and honey bees (Valdes and Scofield 2023). For instance, honey bees and ants remove infected brood, whereas termites consume dead and infected brood to avoid pathogen spreading in the colony (Rothenbuhler 1964; Chouvenc et al. 2008; Sun et al. 2013; Pereira et al. 2020).

Bumble bees can also be infected by various pathogens and parasites, but specific brood diseases, as for honey bees, are unknown. Nevertheless, other parasites, like the microsporidian *Nosema bombi*, have been implicated in the decline of bumble bee populations (Cameron et al. 2011). The trypanosome gut parasite *Crithidia bombi* negatively affects foraging behaviour, growth and survival of colonies, and causes increasing bumble bee mortality (Brown et al. 2000). Hygienic behaviour is rarely described for bumble bees (Valdes and Scofield 2023). For example, Munday and Brown (2018) showed that *Bombus terrestris* remove dead nestmates and dead larvae artificially added to colonies, with removing larvae more rapidly than adult bee corpses. However, specialisation for bumble bee workers performing the observed ‘undertaker’ behaviour was variable (Munday and Brown 2018). Workers more involved in guarding tasks might perform more actively ‘undertaker’ behaviour than nursing workers (Walton et al. 2019).

In the agricultural landscape, foraging bees are exposed to microorganisms that are applied for plant protection. Microbial plant protection products containing the ubiquitous, Gram‐positive bacterium *Bacillus thuringiensis* are used to control pest insects, such as from the order Lepidoptera, Coleoptera, and Diptera (Schnepf et al. 1998; Bravo et al. 2011). *Bacillus thuringiensis* synthesises toxins, such as the highly insecticidal *Cry*‐ and *Cyt*‐toxins (Höfte and Whiteley 1989), causing mortality in target insects (Bravo et al. 2007; Bravo et al. 2011). However, with an increasing use of such plant protection products, there is an increasing chance that non‐target organisms such as pollinators will be exposed too (Babin et al. 2020). After spray application, honey bee and bumble bee workers may collect contaminated nectar and pollen, thereby finally contaminating the whole colony (Alkassab et al. 2022). Honey bee larvae that were exposed to a product containing *B. thuringiensis* ssp. *aizawai* (strain: ABTS‐1857) showed decreased survival rates under laboratory conditions (Steinigeweg et al. 2021). In‐hive feeding revealed that exposed honey bee colonies had higher brood termination rates, and adult bees showed signs of gut microbiome dysbiosis (Steinigeweg et al. 2022). The presence of affected larvae, or at least individuals with a lower chance to survive, may trigger the hygienic behaviour of bumble bees to clear colonies of unhealthy brood. However, targeted brood removal in non‐*Apis* bees has rarely been assessed, especially not for bacteria‐infected brood in bumble bee colonies.

Here, we investigate the response of bumble bees after repeated exposure to a commercial product that contains the bacterium *B. thuringiensis* ssp. *aizawai* (strain: ABTS‐1857). We study the effect on the development of *B. terrestris* colonies as well as the level of hygienic behaviour (characterised by active larvae removal) as a consequence of larval exposure to this microbial agent.

## 2 Materials and Methods

### 2.1 Experimental Design

The experiment took place between April and May 2024 and started with fifteen commercial colonies of the buff‐tailed bumble bee ( *Bombus terrestris* ; Biobest, Belgium), headed by a single queen and compromising of 117 ± 21 bumble bee workers. These colonies were divided into three different treatment groups (C (control), T1 (wounded: artificial manipulation of the brood nest by wounding larvae using a scalpel) or T2 ( *B. thuringiensis* treated, referring to *B. thuringiensis* ssp. *aizawai* (strain: ABTS‐1857)), *n* = 5 colonies per treatment, description of treatment see below), with slightly smaller and larger colonies equally distributed among them, by considering colony's weight, size and number of individuals for standardisation. The colonies were placed in modified wooden honey bee hives with their nesting boxes (w × h × d: 20 cm × 13.5 cm × 26.5 cm), at two different locations at the Julius Kühn Institute (JKI) in Braunschweig, Germany. The C and T1 treatment colonies (coordinates: 52.274573, 10.567465) were placed 270 m apart from the *B. thuringiensis* (T2) treated colonies (coordinates: 52.276839, 10.568684) to avoid potential contamination by drifting workers. A newly developed trapping system, to collect removed dead larvae, was placed in front of the plastic nesting boxes. The trap's design and functionality is comparable to the trapping system for dead worker bees of honey bee colonies (Gary 1960). The trap was built from a wooden box with a metal grid (hole size: 8 × 8 mm) at the top. By leaving the nesting box and leaving the wooden bee hive box for foraging, bumble bee workers had to leave the trap upwards by climbing out and thus were forced to drop everything they may carry out (e.g., a larva). The larva will fell onto a plexiglass plate on the bottom of the trap, which was replaceable, so that larvae could be sampled. Plexiglass plates were replaced daily by clean ones. The surrounding modified honey bee hive allowed bumble bees only to exit through a small hole at the top of the box (Figures S1 and S2).

### 2.2 Hygienic Behaviour and Colony Development

To study exposure associated hygienic behaviour of *B. terrestris* , three groups were set up in the field and were exposed five times (*t* = 0, 7, 10, 15 and 18 days). Time *t* = 0 days was the day of the first exposure. Group C was the non‐exposed control, only receiving 20 mL sugar solution (2 M sucrose) in a 50 mL plastic tube with two feeding holes at the bottom. The first treatment group, T1 (wounding), also received 20 mL sugar solution by spreading the sugar solution equally over the brood nest. For additional brood manipulation a tweezer was used to open the cerumen and underlying brood cells. The brood was randomly scratched with a sharp scalpel. T1 served as a positive control for harmed larvae to trigger larvae removal. In the T2 group, the brood was opened and the nest was exposed to 20 mL of a 2 M sucrose solution spiked with FlorBac (Belchim Crop Protection Germany GmbH). The used concentration of the product was 10% of the maximum field recommended concentration (MFRC) following label instructions, which contains 540 g/kg of the bacterium *B. thuringiensis* ssp. *aizawai* (strain: ABTS‐1857). The 10% spiked sugar solution contains 132 mg test item/kg (Wueppenhorst et al. 2024) corresponding to 7.9 × 10^6^ CFU/g. This concentration reflects the realistic environmental detected CFUs in stored pollen and nectar of honey bees (3.67–3.96 × 10^6^ CFU/g), after application of the product in oilseed rape fields (Alkassab et al. 2022). To ensure the exposure of the brood, the spiked sugar solution was spread equally over the brood nest. Three times a day (9 a.m., 12 noon, 3 p.m.) the plexiglass plates were removed and all removed larvae were sampled. Individual larvae were transferred with sterile tweezer into 4 mL Rotilabo sample tubes each (diameter: 15 mm, Carl Roth). Sample tubes were sealed with a stopper and weighed. The weight of the larva was calculated by subtracting the sample tube empty weight. Larval colour and morphological abnormalities were also noted. All samples were frozen at −80°C.

Colony development was monitored every seven (±2) days (*t* = −11, −4, 2, 9, 15, and 24 days). The first and second assessment occurred before the first exposure. Time t = −11 days was the arrival of the bumble bee colonies at the institute. At this time, all food containers, provided by the supplier, were closed. All colonies were weighed with their nesting boxes, but without the food containers. One day later, all colonies were moved outside to their respective experimental sites. At each assessment the condition of the queen and worker bees was checked visually, dead adults and larvae inside the nesting box were counted and removed. The brood nest stage, brood nest development and cerumen size were also recorded. At later assessment time points, some worker bees sat outside, on the lids of the nesting boxes, and formed so‐called satellite microcolonies. The number of these bees was recorded and the satellite microcolonies were removed. At each assessment, the colonies were photographed to estimate the brood nest size in the nesting box. At the end of the experimental period (*t* = 24 days), the colonies were weighed again to determine weight gain over time.

On day 24, the outdoor part of the experiment was terminated as several newly‐emerged young queens (gynes) were sitting on the lid of the nesting boxes, many colonies had satellite microcolonies on top of the nesting boxes, some colonies were infested with wax moths, and the majority of the colonies only had pupae instead of new larvae. The bumble bees on the lid were mostly gynes and workers. They were collected separately from each colony, counted, weighed and frozen at −20°C. The nesting boxes of the 15 colonies were moved to the laboratory and weighed, and were kept for a further 17 days in the laboratory to allow brood to develop into adult bees. All foraging bumble bees, which were left during moving the colonies to the lab, were collected colony‐wise, counted, weighed and frozen at −20°C the day after migration. These weights were added to the weights at day 24 to get exact final colony weights.

At least once a week, the colonies were fed with a tablespoon of pollen, which was collected using pollen traps at honey bee colonies in spring 2022. Finally, all bumble bee colonies were frozen at −20°C for further inspection. Some colonies (C‐1, C‐2, C‐3 and T1‐10) showed strong damage by wax moths (Table 1) which made estimation of the colony stage complicated at the end of the experiment (Figure S3). After freezing, all bumble bee adult individuals were collected from the nest. These bees, and also the individuals collected separately, were counted and their sex was determined to obtain colony specific sex ratios. Under natural conditions, the sex ratio of queens to drones should be on the drones' side (Beekman and van Stratum 1998; Beekman and van Stratum 2000). The sex ratio was calculated using the formula drones/(drones+queens) (Beekman and van Stratum 2000).

**TABLE 1:** Summary of colony development and specific colony traits, including the numbers of workers, queens and drones per colony, sex ratio, the total number of removed larvae, colony weight gain, colony size until day 15, and the estimated wax moth damage on day 24. The sex ratio was calculated using the formula drones/(queens+drones). If the sex ratio is > 0.5 the colony is drone‐dominated; if the sex ratio is < 0.5 the colony is queen‐dominated. Day 0 was the day of the first exposure, on day 15 was the maximum reached in colony size, and day 24 was the termination of the experiment (C: Control, T1: Wounded, T2: *B. thuringiensis* treatment).

Colony number	Treatment	No. of workers	No. of queens	No. of drones	Sex ratio	Total no. of removed larvae	Colony weight gain (g)	Colony size (cm^3^)	Wax moth damage (%)
1	C	116	6	7	0.54	10	455	3832	100
2	C	75	7	18	0.72	15	250	3317	100
3	C	115	41	24	0.37	6	226	2404	100
4	C	181	42	101	0.71	4	308	3459	5
5	C	72	36	59	0.62	24	491	5676	0
6	T1	136	56	48	0.46	6	197	3236	25
7	T1	117	30	30	0.50	7	255	2968	50
8	T1	127	52	32	0.38	9	289	3945	0
9	T1	309	42	36	0.46	2	276	3601	5
10	T1	320	60	52	0.46	28	647	5087	100
11	T2	116	43	105	0.71	24	558	3616	0
12	T2	108	88	88	0.50	15	424	4346	0
13	T2	131	58	130	0.69	22	321	2698	0
14	T2	106	29	194	0.87	16	431	5309	0
15	T2	118	18	18	0.50	11	231	2177	0

### 2.3 Detection of *Bacillus thuringiensis*

A total of 73 larvae were tested for the presence of *B. thuringiensis* , with 22 larvae from the control colonies, 18 from the wounded colonies (T1), and 33 larvae from the *B. thuringiensis* treated (T2) colonies. The larvae used for exposure verification were the ones that were removed by the bumble bee workers 1 or 2 days after in‐colony exposure. Each larva was homogenised in 200–800 μL ddH_2_O (depending on the weight of the larva) in 1.5 mL reaction tubes using sterile pestles. For the following procedure, aliquots of larvae homogenates from the control group were pooled when they were removed on the same day of the experiment, as it was assumed that these larvae were not infected. Aliquots of larvae homogenates of the wounded group (T1) were also pooled for the same reason. Larvae from *B. thuringiensis* treated colonies (T2) were tested individually and sorted by colour. Finally, 50 samples (pools and single larvae) were tested. Each larvae homogenate was diluted down to 10^−6^. Serial dilutions were plated on sterile LB agar plates (with 100 mg/L penicillin to select for *B. thuringiensis* ssp. *aizawai* (strain: ABTS‐1857) due to its known penicillin resistance, Anastassiadou et al. 2020) in duplicates and incubated at 30°C for 24 h. For the positive control, 10 μg FlorBac was dissolved in 200 μL ddH_2_O, diluted to 10^−6^, and cultivated, not in duplicates, on LB agar + penicillin plates.

Grown bacterial colonies of different colour, size and shape were picked sterile in 50 μL ddH_2_O and stored at −20°C. One colony was taken from the positive control. These samples were used for PCR to confirm their species identity with *B. thuringiensis* . The PCR was performed according to Wei et al. (2019) using 84 picked colonies (C: 13, T1: 5 and T2: 66 colonies) and a respective positive control. PCR products (expected size: 246 bp) were visualised with Midori Green Advance (Biozym) after gel electrophoresis (1% agarose), using GeneRuler 1 kb Plus DNA Ladder (Thermo Fisher Scientific) as marker.

### 2.4 Data Analysis

Photographs of bumble bee colony development were analysed using ImageJ (version 1.54i). The number of individuals was counted using the ImageJ function Cell Counter and the size of the brood nest was measured using the ImageJ function Measure.

On some days, few colonies removed significantly more larvae than on other days. These outliers were not used in the statistical analyses and figures (Figure S4). Statistical analyses were performed using standard spreadsheet software and R (version 4.4.0). Normal distribution was tested with a Kolmogorov–Smirnov test. Sex ratios were compared pair‐wise with two‐sample *t*‐tests and Bonferroni correction for multiple testing. Correlation analyses were performed to test for (1) a relationship between the sex ratio and colony size or weight (using the final weight determined before wax moth damage), and (2) a relationship between larvae removal and nest size (on day 15) or colony weight. Differences in larvae removal over time and between treatment groups was analysed using linear mixed effects modelling. Therefore, the *glmmTMB* function of the *glmmTMB* package (version 1.1.10) was used (Brooks et al. 2017) based on a Poisson distribution. Larvae removal was tested with an interaction between treatment group and time as fixed factors and colony number as random effect. Estimated marginal means of model output were compared using the *emmean* function of the *emmeans* packages (version 1.8.4–1) (Lenth 2023) and a Bonferroni adjustment for multiple testing. Given the high variance among colonies, the model was not perfectly fitting observed and expected values in the QQ plot (Kolmogorov–Smirnov test *p* < 0.05, dispersion test *p* > 0.05, outlier test *p* > 0.05) following visual evaluation, but the best model to analyse the data.

## 3 Results

### 3.1 Hygienic Behaviour

**FIGURE 1:**
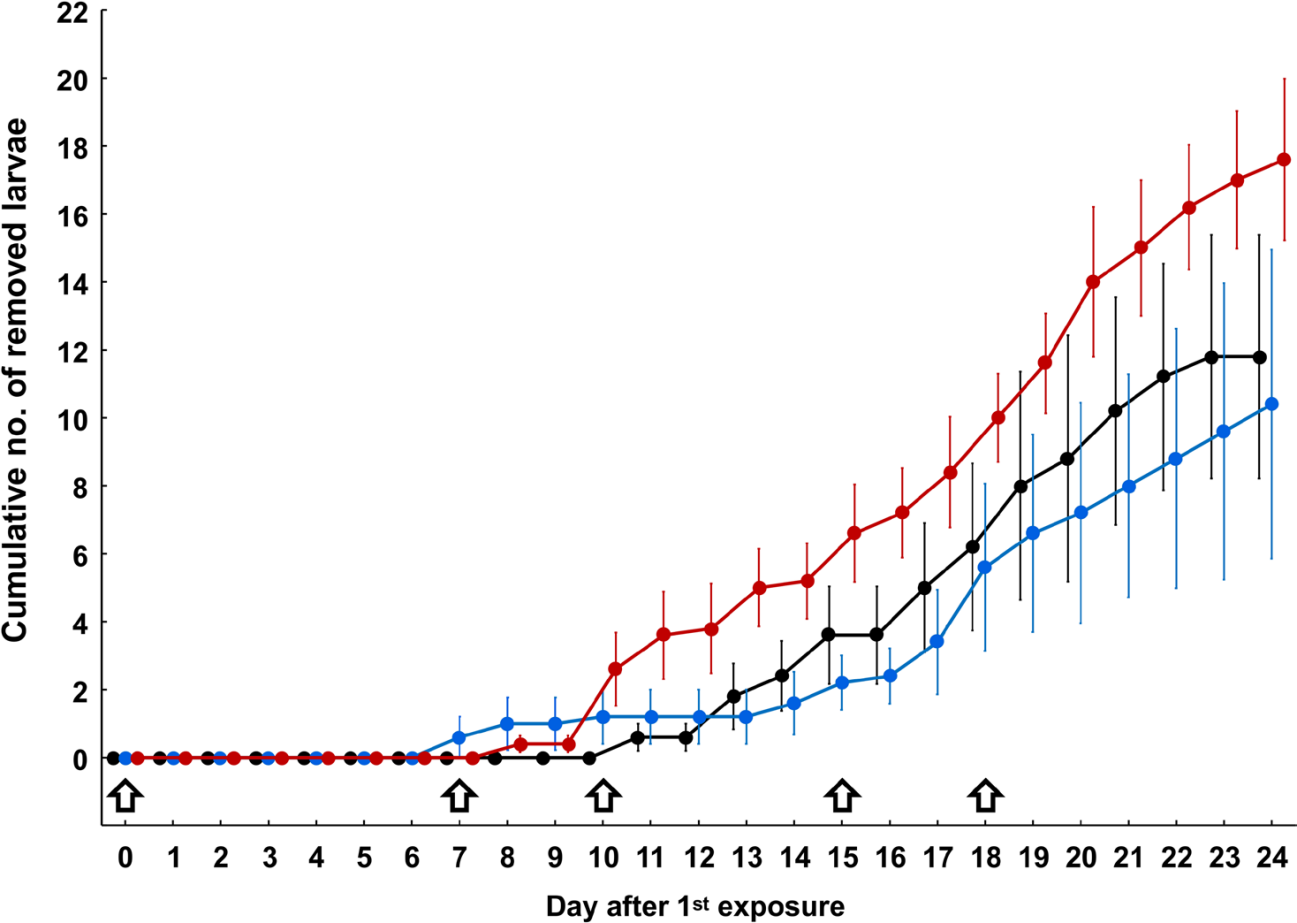
Development of larval removal over time. Shown are the average cumulative numbers of larvae removed from all colonies in each treatment group (*n* = 5 per group) ± SE. Arrows indicate the days of exposures. (black: Control colonies, blue: T1 ‐ wounded colonies, red: T2 ‐ *B. thuringiensis* treated colonies).

Larvae removal began on the day of the second exposure, 7 days after the first exposure, with T1 (wounded) colonies showing this specific behaviour (Figure 1). The next day, *B. thuringiensis* treated colonies (T2) started to remove larvae, followed by the control colonies, which started on day 11 after the first exposure. At the end of the observation period, *B. thuringiensis* treated colonies (T2) removed the most larvae, with an average of 17.6 larvae (±5.3 SD) per colony over the whole period. The control colonies removed on average 11.8 larvae (±8) and wounded colonies (T1) removed the fewest 10.4 (±10.2) (LMM: C vs. T1—*p* = 0.84, C vs. T2—*p* = 0.009) (Figure 1).

Comparing larvae removal with colony size (in cm^3^) on day 15 revealed a marginally significant correlation (*r* ^2^ = 0.26, *p* = 0.054) (Figure S5). The largest nest (colony 5), with a volume of > 5600 cm^3^, had a high number of removed larvae (24 larvae), and the smallest nest (colony 15), with a nest size of 2177 cm^3^, had a low number of removed larvae (11 larvae) (Table 1). Larvae removal correlated strongly with colony weight gain (in g) (*r* ^2^ = 0.61, *p* = 0.0006) (Figure S5). Many colonies with a low weight increase removed only a few larvae, whereas many colonies with a high weight increase removed more larvae (Table 1).

**FIGURE 2:**
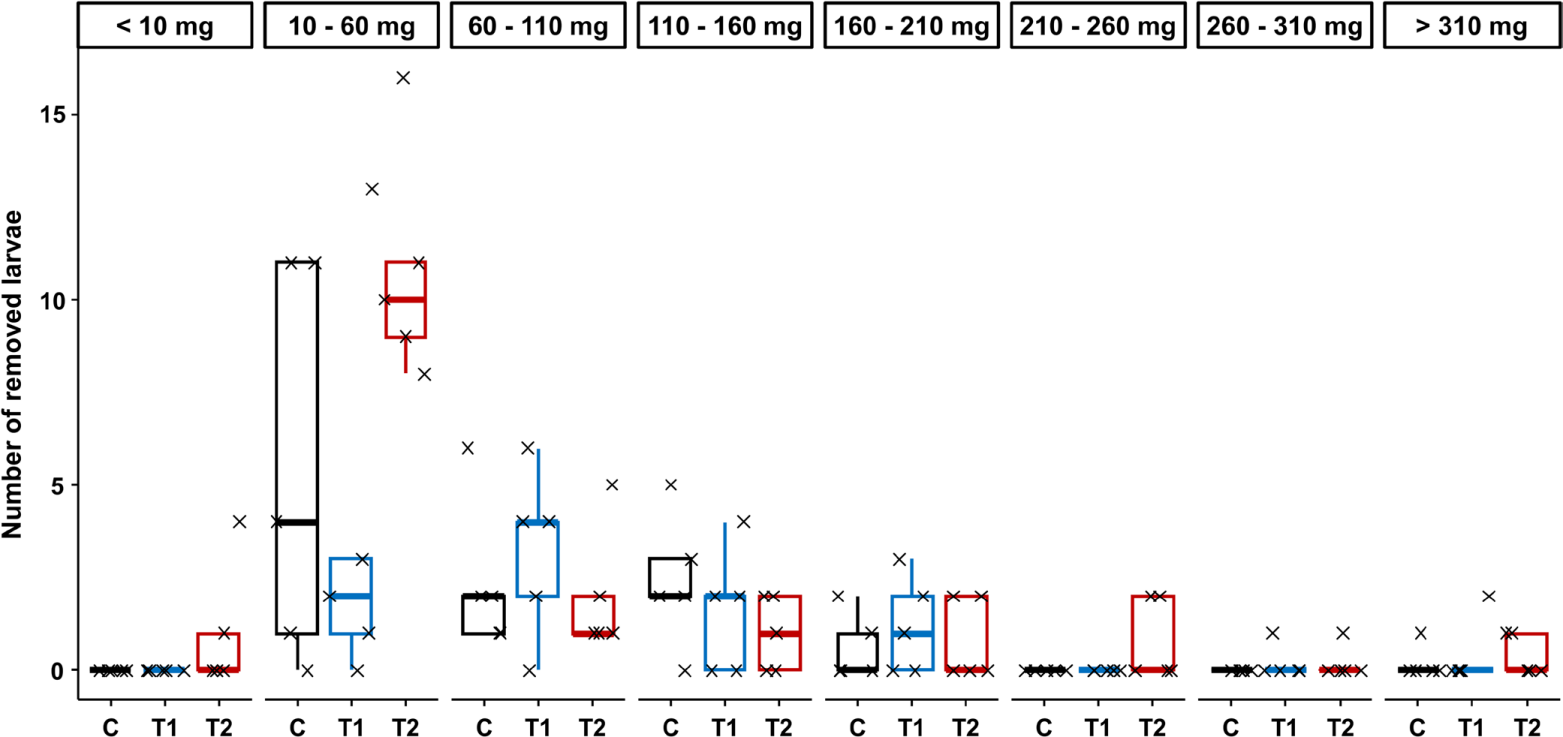
Removed larvae per weight category and treatment group (*n* = 5 per group). Crosses show the total number of larvae removed per colony. Box plots show the medians and interquartile ranges. (C: Control—black, T1: Wounded—blue, T2: *B. thuringiensis* treated—red).

To get an overview on size distribution of removed larvae, larvae were sorted into different weight categories. Most of the removed larvae (*n* = 103) weighed between 10 and 60 mg, with slightly more than half of the larvae belonging to the *B. thuringiensis* treatment (T2). Very few larvae were lighter than 10 mg or heavier than 210 mg, again dominated by the *B. thuringiensis* treatment. No control colony removed any larvae of these extreme weight categories (Figure 2).

**FIGURE 3:**
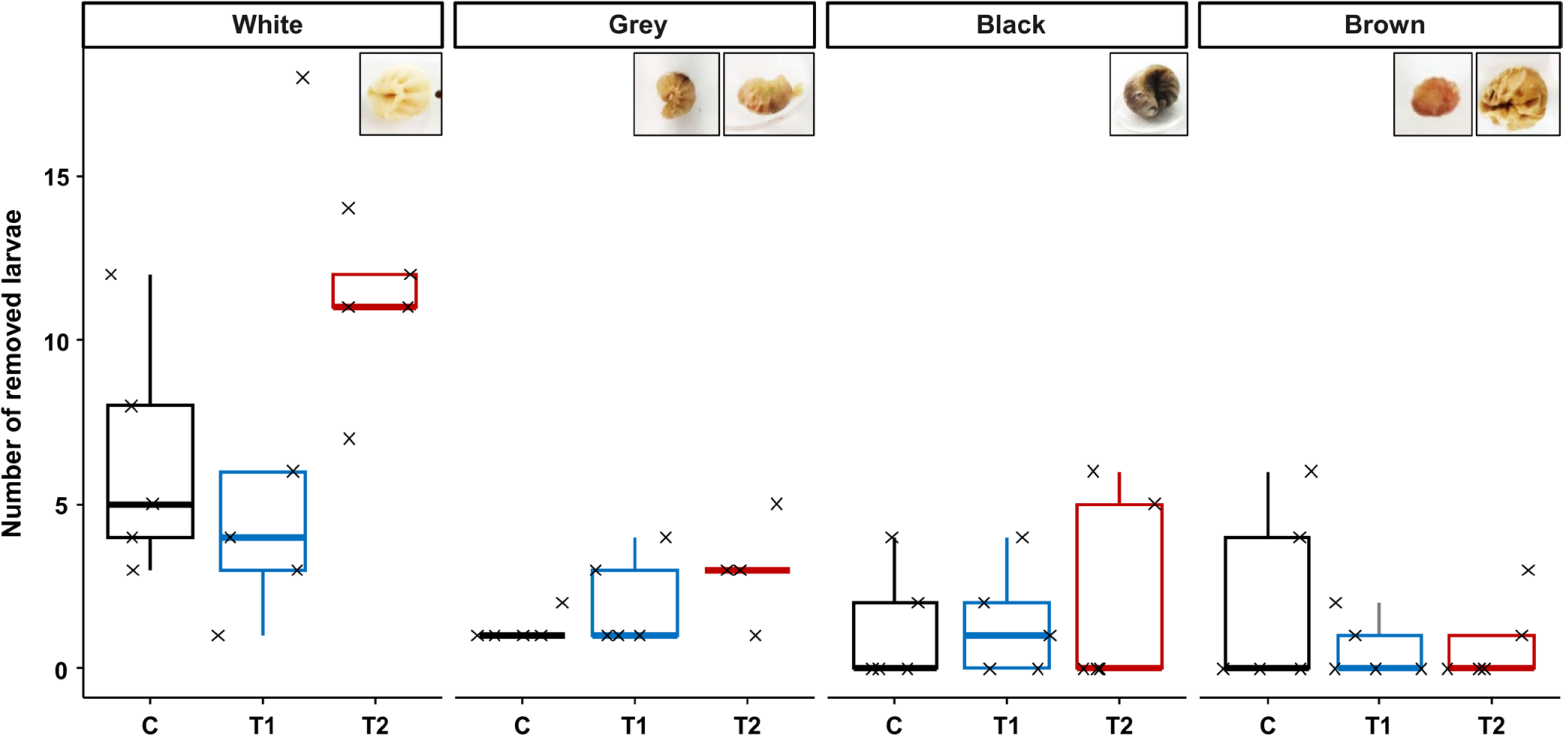
Removed larvae per colour category and treatment group (*n* = 5 per group). Crosses show the total number of larvae removed per colony. Box plots show the medians and interquartile ranges. (C: Control—black, T1: Wounded—blue, T2: *B. thuringiensis* treated—red).

When looking at the colour of the removed larvae it became evident that predominantly white larvae were removed, with the highest number of white larvae being removed in the *B. thuringiensis* treated colonies (T2). Grey and black larvae were mostly sampled from wounded (T1) and *B. thuringiensis* treated colonies. The control colonies removed the largest proportion of brown larvae (Figure 3).

**FIGURE 4:**
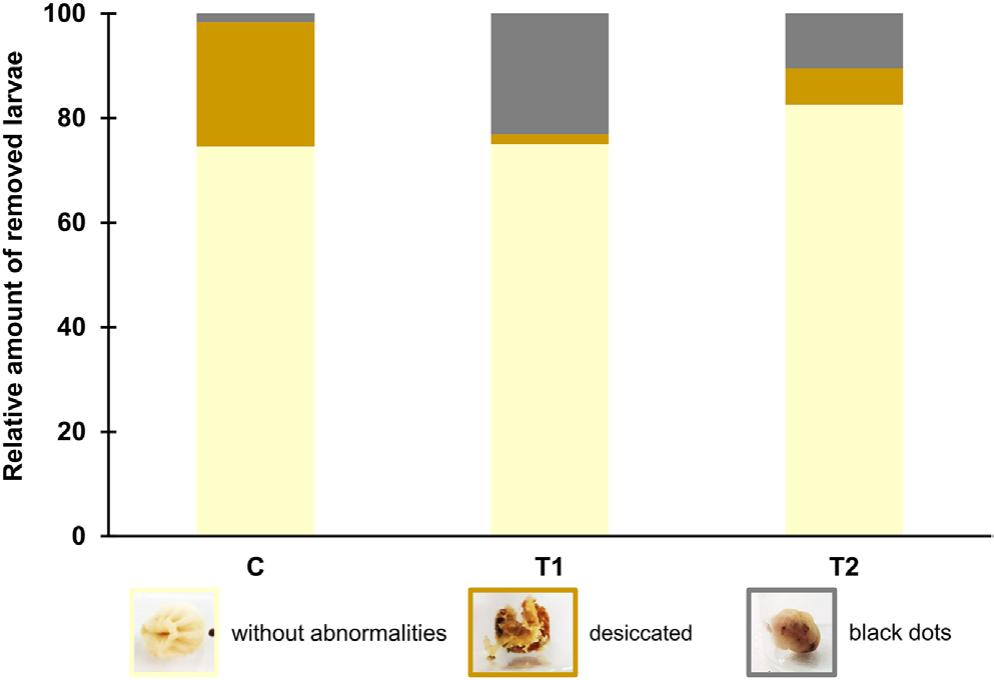
Removed larvae per treatment group and observed abnormalities (no abnormalities—yellow, desiccated—brown and larvae with black dots—grey). Representative bumble bee larvae are shown for all categories.

Most of the larvae had no abnormalities (> 70% of each treatment group), but some showed signs of black dots or they were desiccated. The comparison among treatments showed that control colonies exhibited the highest proportion of desiccated removed larvae (24%) and T1 (wounded) colonies the highest share of larvae with black dots (23%). *Bacillus thuringiensis* treated colonies (T2) removed larvae with black dots and signs of desiccation at rates of 7%–11% (Figure 4).

The bacteria‐specific PCR results confirmed that the tested removed larvae from control (*n* = 22) and T1 (wounded) (*n* = 18) colonies were free of *B. thuringiensis* . Approximately one third of the tested larvae from the *B. thuringiensis* treated colonies (T2) were positive for *B. thuringiensis* (Table 2a). The bacterial colonies selected from respective agar plates of the larvae tested from bumble bee colony 15 showed no positive result for the *B. thuringiensis* PCR (Table 2b). The majority of *B. thuringiensis‐*positively tested larvae showed a larval weight between 10 and 60 mg (Table S1A) and most of the exposed and removed larvae were white (Table S1B).

**TABLE 2:** Detection of *B. thuringiensis* in *B. terrestris* larvae. The number of larvae tested for *B. thuringiensis* and the number of *B. thuringiensis* positive larvae per treatment group (A) and colony for T2 colonies (B) (C: Control, T1: Wounded, T2: *B. thuringiensis* treated).

A		
Treatment	No. of tested larvae	No. of *B. thuringiensis* positive larvae
C	22	0
T1	18	0
T2	33	12

B		
Colony	No. of tested larvae	No. of *B. thuringiensis* positive larvae
T2‐11	7	3
T2‐12	6	1
T2‐13	10	4
T2‐14	6	4
T2‐15	4	0

### 3.2 Colony Development

Comparing the sex ratios among treatments, the control colony's sex ratio was mostly drone‐dominated. Only in one case the ratio was dominated by queens (colony 3: sex ratio of 0.37) (Table 1). All T1 (wounded) colonies had a ratio of ≤ 0.5, which means they were queen‐dominated, except for colony 7 with a value of 0.5. Most of the colonies treated with *B. thuringiensis* (T2) had a drone‐dominated sex ratio, only colony 12 and colony 15 had the same number of queens and drones. Finally, the sex ratios of the different treatment groups did not differ from each other (*t*‐test—C vs. T1: *t* = 2.08, df = 8, *p* = 0.071; C vs. T2: *t* = −0.65, df = 8, *p* = 0.53; T1 vs. T2: *t* = −2.77, df = 8, *p* = 0.024, adjusted significance level *p*_adj_ = 0.017).

Neither colony weight gain (*r* ^2^ = 0.07, *p* > 0.05) nor colony size (*r* ^2^ = 0.08, *p* > 0.05) correlated with the observed sex ratios. However, queen‐dominated wounded colonies (T1) had the lowest average weight gain, and the drone‐dominated *B. thuringiensis* treated colonies (T2) showed the largest weight gain.

## 4 Discussion

A recent study on honey bees showed that the number of removed larvae increased with increasing nest size, as larger colonies exhibit stronger hygienic behaviour (Snyder et al. 2023). In the current study, using the bumble bee *B. terrestris* , a positive trend between colony size and larvae removal could be observed too, even though it was only significant when testing colony weight and larvae removal. One possible explanation might be that with increasing colony size more workers have built satellite microcolonies on the lids of the nesting boxes, leaving fewer workers in the colony to remove larvae. Stress induced by colony handling or the high density of worker bees in the nesting boxes might be factors initiating microcolony formation. With workers leaving the main nest for microcolonies, the number of drones increases in the nesting box. As drones do not show hygienic behaviour, the decreasing number of workers will result in lower numbers of removed larvae at this time. An alternative hypothesis might be the timing of reaching the competition point, the stage of colony development where queens and workers compete for laying drone eggs, which affects the colony's future (Alaux et al. 2006; Duchateau and Velthuis 1988). An early competition point results in more drones, while a later one results in more gynes and a larger colony. However, also the timing of the switching point needs to be considered, as the time point where queens switch from laying diploid eggs to haploid drone eggs (Duchateau and Velthuis 1988). Nevertheless, from day 10 onwards, the *B. thuringiensis* treated colonies removed the highest number of larvae. PCR analysis revealed the presence of *B. thuringiensis* in some of the removed larvae of the respective treatment group, but not in the wounded or control colonies. This is in accordance with the results found in a previous study (Schuehly et al. 2021). They showed that honey bee workers can recognise diseased larvae and activate specific behavioural responses, such as larvae removal following pesticide (clothianidin) and lipopolysaccharide exposure.

Most of the removed bumble bee larvae had a light weight of 10–60 mg and only a few weighted more than 160 or 210 mg, respectively. It is well known from honey bees that larvae in an early stage are more vulnerable to bacterial infections than those at later stages (Hoage and Rothenbuhler 1966). This might have resulted in the higher number of removed larvae weighing between 10 and 60 mg in *B. thuringiensis* treated colonies, which might also be more sensitive to *B. thuringiensis* . Early larval stages of honey bees also showed to be more susceptible to *B. thuringiensis* compared to later brood stages (Steinigeweg et al. 2021). Visual changes (colour or texture) related to larvae infections has already been described for diseased stingless bees and honey bees, in the worst case being brown or black, desiccated or liquefied (Shanks et al. 2017; Kathe et al. 2021). Here, the majority of the removed larvae were white with no abnormalities in texture. Nevertheless, visual parameters may indicate how long an infection exists, and with observing an altered appearance that may also indicate sickness in bumble bee larvae. As larvae colour change seemed to be only a limited indicator of sickness after exposure to *B. thuringiensis* (Figure 3 and Table S1B), it is possible that differently coloured larvae might be explained by other biotic or abiotic factors (not tested in the current study). The same phenomenon was observed under field conditions, where bumble bee workers foraged freely in FlorBac treated oilseed rape fields and workers removed white larvae as well as differently coloured larvae, which were found outside the bumble bee nesting boxes (Alkassab and Wueppenhorst, pers. observation, Figure S6 and Video S1).

As already mentioned, exposure to *B. thuringiensis* resulted in increasing hygienic behaviour in *B. terrestris* colonies. Until now, hygienic behaviour has not been widely studied or detected in bumble bees, like in termites, ants and honey bees (Valdes and Scofield 2023). For example, infections with *N. bombi* did not result in higher larvae removal in *B. terrestris* microcolonies, compared to healthy controls (Munday and Brown 2018). The authors concluded that they did not detect evidence for prophylactic removal to protect the colony from disease spread. On the other hand, larvae removal or ejection from bumble bee microcolonies was observed frequently using different pollen sources, pollen mixes, control diets or supplemented diets for studying colony development (Génissel et al. 2002; Tasei and Aupinel 2008; Gekière et al. 2022). However, these might be cases of malnutrition, nutrient‐limitation, intoxication with plant secondary metabolites or other nutrient‐related traits and may not directly be related to hygienic behaviour. Additional infections with *C. bombi* did not result in differences of larvae ejection rates (Gekière et al. 2022). Based on the current study and existing literature on bees, ants and termites, hygienic behaviour seems to be a fundamental part of social living in (eu)social insects, protecting their colonies and nests from disease spread and securing colony fitness. Even facultatively eusocial bees show behaviour of dead brood removal from their nests (Michener 1972). Also, foundresses of the social paper wasp *Polistes dominula* selectively remove biopesticide‐based microorganism exposed larvae (Cappa et al. 2024). Comparing the different levels of sociality and life cycles of (eu)social insects, perennial colonies may have a stronger pressure for long‐term nest hygiene than annual colonies of social organisms (Zhu and Wang 2026). Based on the results of the current study, bumble bees add an essential piece to the puzzle of understanding the importance of the central role of hygienic behaviour for colony functioning, including nest sanitation and brood regulation. Hygienic behaviour, the removal of diseased larvae or pupae, is discussed to be associated with: (1) certain volatile chemical compounds that are not present in healthy larvae or (2) bouquet changes or (3) larvae will be removed that do not smell ‘normal’ (Kathe et al. 2021). For example, worker honey bees can recognise such compounds with their antennae, which triggers the activation of hygienic behaviour (Rothenbuhler 1964). Further research is needed to understand how the observed behaviour and methods of detecting affected nestmates and larvae are conserved among different bumble bee species.

The majority of the control and *B. thuringiensis* treated colonies were drone‐dominated, whereas the wounded colonies were rather queen‐dominated. This suggests that due to brood nest manipulation more gynes hatched than drones, as maybe more drone larvae were wounded and finally died. As already mentioned, the timing of the switching point and competition point might also be central factors impacting the sex ratio of the colonies (Duchateau and Velthuis 1988). However, as there was no significant difference between treatments, neither the treatment with the plant protection product containing *B. thuringiensis* nor the brood nest manipulation (larvae wounding) had a strong impact on the production of drones and gynes. On average, the sex ratio favoured the drones (0.57), as it was expected from literature. However, these values were still lower than the values of 0.61–0.94 reported by Beekman and van Stratum (1998). Another major factor impacting the sex ratio might be the brood nest damage caused by the wax moth larvae and processes of colony decay. Consequently, the exact number of queens, drones and workers was impaired as well as the general development of the colonies. Only colonies exposed to *B. thuringiensis* were not damaged as the used *B. thuringiensis* strain is a pathogen and kills Lepidoptera larvae, including wax moths (Bravo et al. 2011). On the other hand, *B. thuringiensis* affected brood development of honey bees ( *Apis mellifera* ), with significantly fewer workers hatching (Steinigeweg et al. 2022). However, due to wax moth damage to the control and wounded colonies at a later stage of the experiment, it is not possible to conclude whether *B. terrestris* brood nest development was also affected by *B. thuringiensis* . Nevertheless, the current study provides first insights regarding bacteria exposure related hygienic behaviour in bumble bee colonies. Future studies may investigate if there are sex‐specific differences in larvae removal to better understand potential effects on brood nest development.

## 5 Conclusion

The exposure to a plant protection product containing *Bacillus thuringiensis* ssp. *aizawai* (strain: ABTS‐1857) induced hygienic behaviour of *Bombus terrestris* colonies. More larvae were removed following exposure to this bioinsecticide than in the control group, and larger colonies removed more larvae. Having strong differences in larvae removal between controls and the *B. thuringiensis* exposed colonies indicates that larvae ejection was not a direct stress response due to the general experimental conditions, as colony handling was the same for all treatment groups. Many of the removed larvae had a low weight, indicating that those early stages were particularly more susceptible to exposure and had a higher mortality rate. Larvae colour was not a reliable indicator of exposure to *B. thuringiensis* and potential consequences for the larvae, as white larvae were also exposed and removed. The successful exposure of the mostly white larvae was confirmed by the presence of *B. thuringiensis* but the infection mechanisms are still unclear. These results show that the use of bioinsecticides containing *B. thuringiensis* can affect the brood of *Bombus terrestris* —in particular brood removal, so more research is needed concerning the details of brood nest development, the health of bumble bees and to understand underlying chemical mechanisms, and thus include this knowledge to the ecological risk assessment of plant protection products.

## Author Contributions

**Michelle Scheffler:** data curation (lead), formal analysis (equal), investigation (equal), visualization (equal), writing – original draft (lead), writing – review and editing (equal). **Karoline Wueppenhorst:** conceptualization (equal), formal analysis (equal), investigation (equal), visualization (equal), writing – review and editing (equal). **Doreen Babin:** investigation (equal), methodology (equal), resources (equal), writing – review and editing (equal). **Abdulrahim T. Alkassab:** conceptualization (equal), methodology (equal), writing – review and editing (equal). **Silvio Erler:** conceptualization (equal), data curation (equal), formal analysis (equal), supervision (lead), visualization (equal), writing – original draft (equal), writing – review and editing (equal).

## Funding

We acknowledge the financial support by the Federal Ministry of Agriculture, Food and Regional Identity for funding initial research in this field. Open Access funding enabled and organised by Projekt DEAL.

## Conflicts of Interest

The authors declare no conflicts of interest.

## Supporting information


**Video S1:** Worker bumble bee ( *B. terrestris* ) removing a large white bumble bee larva from its colony (Wueppenhorst, pers. observation, 2021).

**Figure S1:** Trapping system. (A) Front view. There is a slot at the bottom, where a plexiglass shield can be inserted and replaced. (B) Back view. There is a hole in the middle of the mesh fabric. Through the hole the bumble bees can leave their nesting box and directly enter the death trap, dropping larvae before leaving the trap.
**Figure S2:** View inside the wooden bee hive. (A) Top view on the bumble bee nesting box (right). When bumble bees leave the nest, they fly into the trapping system (left). To get out of the trap, they have to climb through the grid. On the far left (black dot) is the exit of the bumble bee nesting box. (B) Side view of a bumble bee nesting box connected to the death trap. At the bottom of the death trap is a removable plexiglass shield.
**Figure S3:** Development of colony 2 nest size over the experimental period. (A) The colony on the arrival day (*t* = −11 days). There is no cerumen present. Only some brood cells exist. (B) Four days before the first exposure (*t* = −4 days). The cerumen covers about the half of the brood nest. More brood cells are clearly visible. (C) Maximum nest size on day 15 (*t* = 15 days). The cerumen covers nearly the entire brood nest. The nest has clearly grown in width and height. In the bottom right corner are many dead larvae and adults, as well as faeces. (D) Wax moth damage (100%). The brood nest has been extensively damaged by wax moths. There is no cerumen present.
**Figure S4:** Larvae removal by larval weight per colony. The number of larvae removed per colony is divided into different size categories. (A) with statistical outliers and (B) without statistical outliers. (C: control, T1: wounded, T2: exposure to *B. thuringiensis* , 1–15: colony number, see Table 1).
**Figure S5:** Relationship between number of removed bumble bee larvae per colony (*n* = 15 colonies) and colony weight gain (in g, left y‐axis, filled circles and solid line, *r* ^2^ = 0.6086, *p* = 0.0006) as well as colony size on day 15 (in cm3, right y‐axis, empty circles and dashed line, *r* ^2^ = 0.2564, *p* = 0.0541).
**Figure S6:** Differently coloured ejected larvae and of different size outside the bumble bee nesting box. This phenomenon was observed under field conditions where bumble bee workers foraged freely in FlorBac treated oilseed rape fields (Alkassab and Wueppenhorst, pers. observation, 2021).
**Table S1:** PCR results grouped by larval weight and colour for the T2 (bacteria exposure) treatment. (A) Number of larvae in the different weight categories tested for *B. thuringiensis* and the number of *B. thuringiensis* positive larvae. (B) Number of larvae in the different colour categories tested for *B. thuringiensis* and the number of *B. thuringiensis* positive larvae.

## Data Availability

The data were deposited at Open Agrar https://www.openagrar.de/receive/openagrar_mods_00112026.
