# Supplementary material for: Microbial Pressure and Social Immunity: Bumble Bees Increase Brood Hygiene After Exposure to a Bacillus thuringiensis ‐Based Biopesticide
Source: Ecol Evol. 2026 Jun 11;16(6):e73844. doi: 10.1002/ece3.73844 (PMC13255005; doi:10.1002/ece3.73844)
Supplement: Supplementary file 2 — Figure S1: Trapping system. (A) Front view. There is a slot at the bottom, where a plexiglass shield can be inserted and replaced. (B) Back view. There is a hole in the middle of the mesh fabric. Through the hole the bumble bees can leave their nesting box and directly enter the death trap, dropping larvae before leaving the trap. Figure S2: View inside the wooden bee hive. (A) Top view on the bumble bee nesting box (right). When bumble bees leave the nest, they fly into the trapping system (left). To get out of the trap, they have to climb through the grid. On the far left (black dot) is the exit of the bumble bee nesting box. (B) Side view of a bumble bee nesting box connected to the death trap. At the bottom of the death trap is a removable plexiglass shield. Figure S3: Development of colony 2 nest size over the experimental period. (A) The colony on the arrival day (t = −11 days). There is no cerumen present. Only some brood cells exist. (B) Four days before the first exposure (t = −4 days). The cerumen covers about the half of the brood nest. More brood cells are clearly visible. (C) Maximum nest size on day 15 (t = 15 days). The cerumen covers nearly the entire brood nest. The nest has clearly grown in width and height. In the bottom right corner are many dead larvae and adults, as well as faeces. (D) Wax moth damage (100%). The brood nest has been extensively damaged by wax moths. There is no cerumen present. Figure S4: Larvae removal by larval weight per colony. The number of larvae removed per colony is divided into different size categories. (A) with statistical outliers and (B) without statistical outliers. (C: control, T1: wounded, T2: exposure to B. thuringiensis , 1–15: colony number, see Table 1). Figure S5: Relationship between number of removed bumble bee larvae per colony (n = 15 colonies) and colony weight gain (in g, left y‐axis, filled circles and solid line, r 2 = 0.6086, p = 0.0006) as well as colony size on day 15 (in cm3, right y‐axis, empty [file ECE3-16-e73844-s001.pdf]

<u>**Supplementary material**</u>

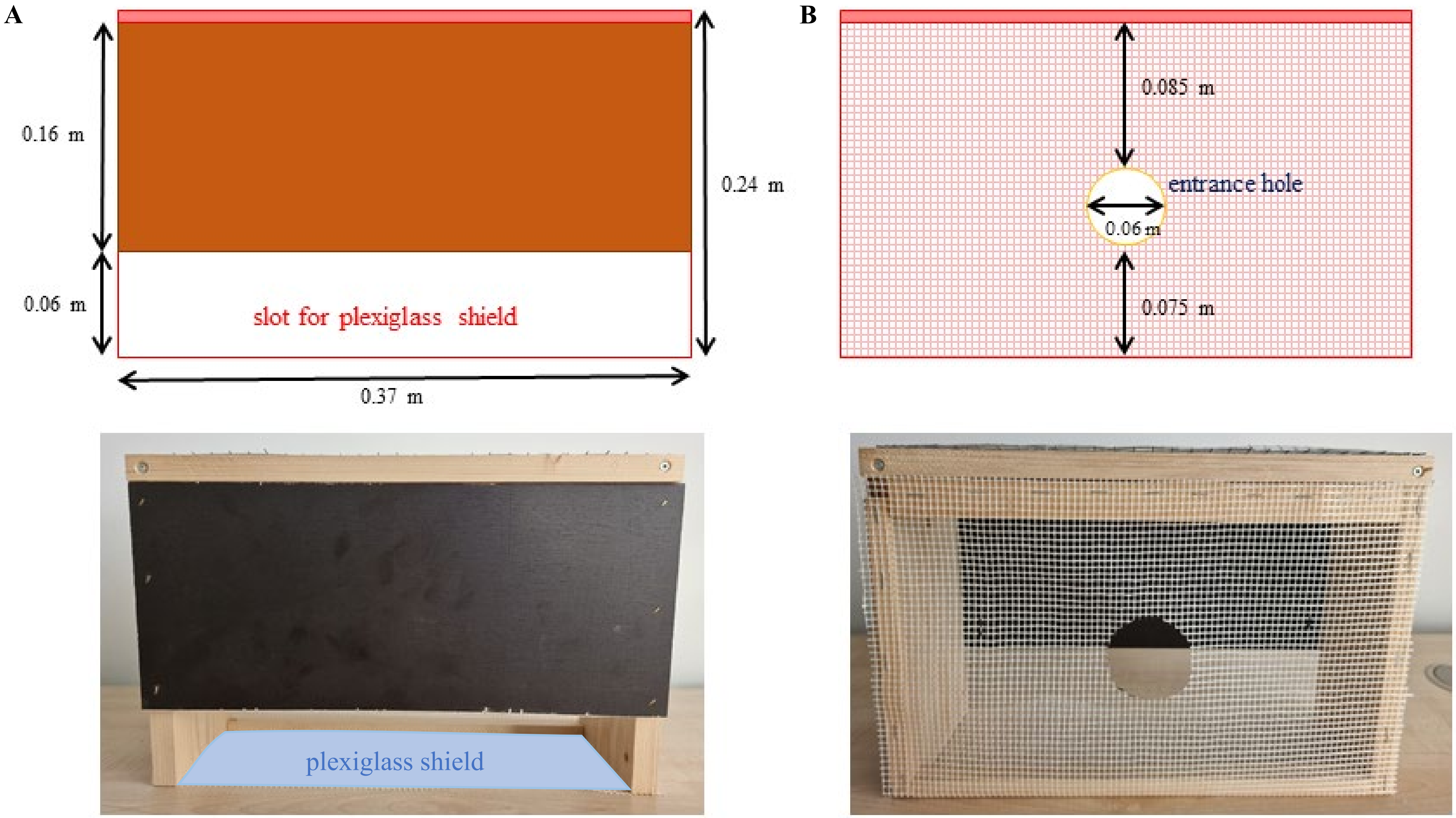

Fig. S1. Trapping system. (A) Front view. There is a slot at the bottom, where a plexiglass shield can be inserted and replaced. **(B) Back view.** There is a hole in the middle of the mesh fabric. Through the hole the bumble bees can leave their nesting box and directly enter the death trap, dropping larvae before leaving the trap.

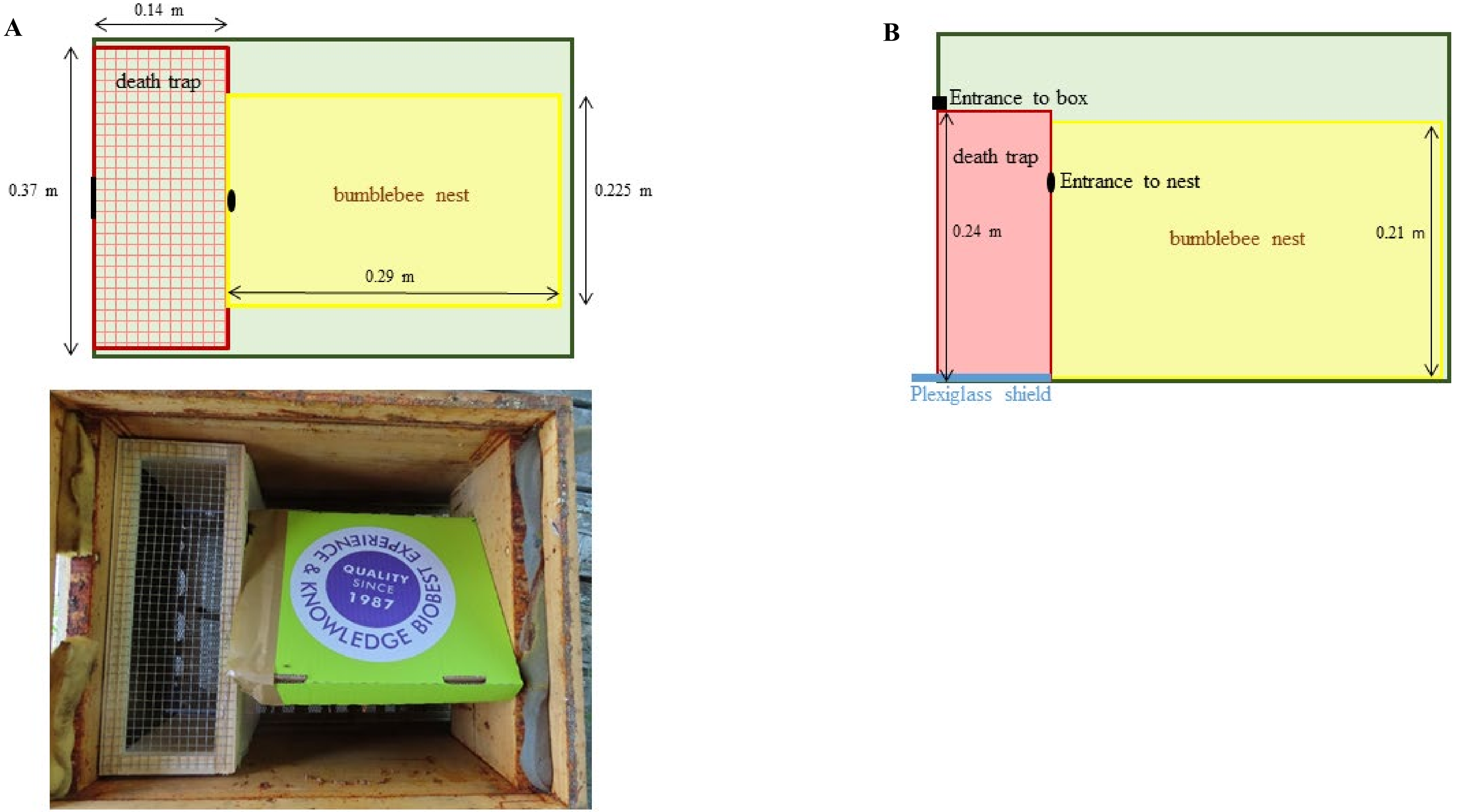

Fig. S2. View inside the wooden bee hive. (A) Top view on the bumble bee nesting box (right). When bumble bees leave the nest, they fly into the trapping system (left). To get out of the trap, they have to climb through the grid. On the far left (black dot) is the exit of the bumble bee nesting box. **(B)** Side view of a bumble bee nesting box connected to the death trap. At the bottom of the death trap is a removable plexiglass shield.

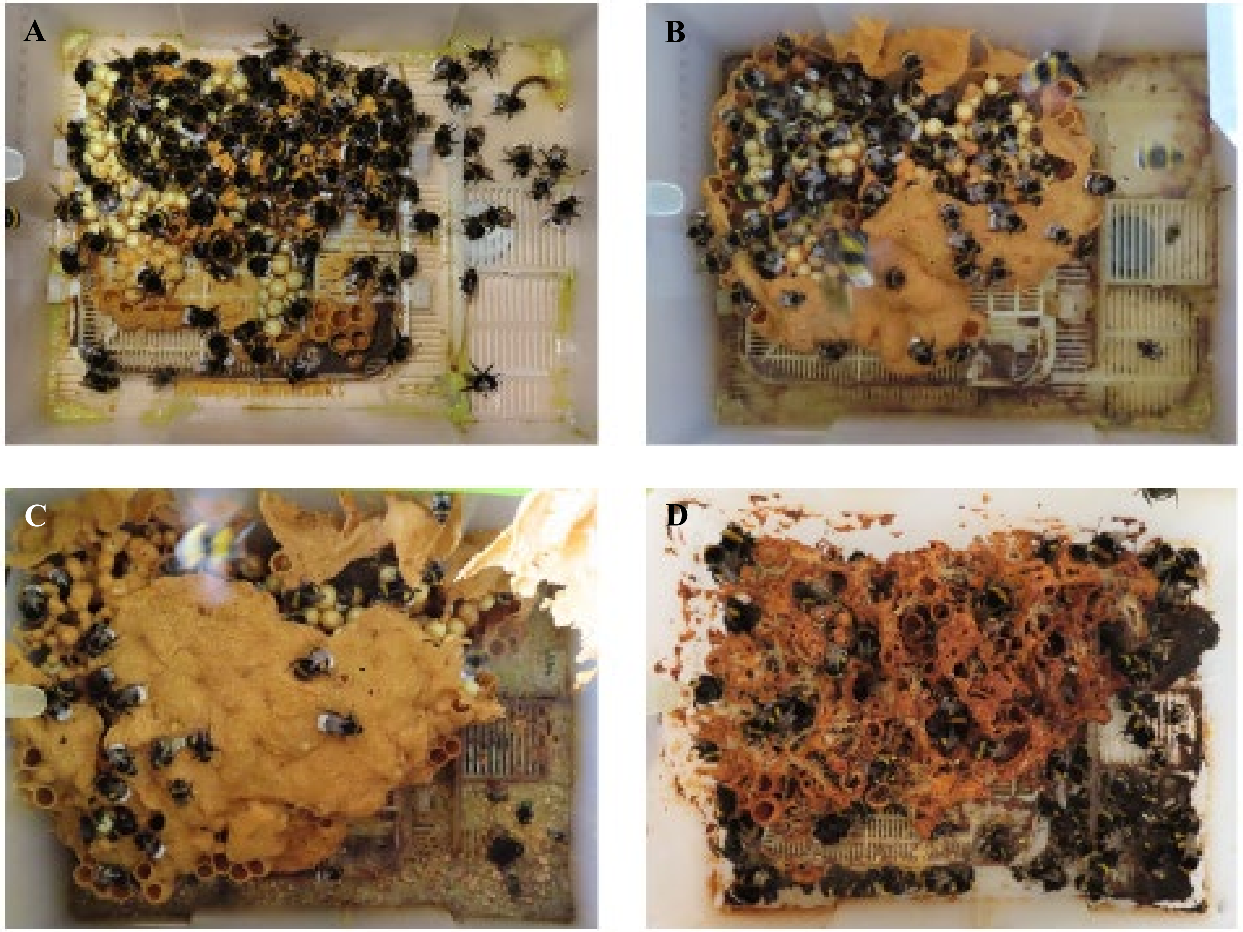

Fig. S3. Development of colony 2 nest size over the experimental period. (A) The colony on the arrival day (t = -11 d). There is no cerumen present. Only some brood cells exist. **(B)** Four days before the first exposure (t = -4 d). The cerumen covers about the half of the brood nest. More brood cells are clearly visible. **(C)** Maximum nest size on day 15 (t = 15 d). The cerumen covers nearly the entire brood nest. The nest has clearly grown in width and height. In the bottom right corner are many dead larvae and adults, as well as faeces. **(D)** Wax moth damage (100%). The brood nest has been extensively damaged by wax moths. There is no cerumen present.

A

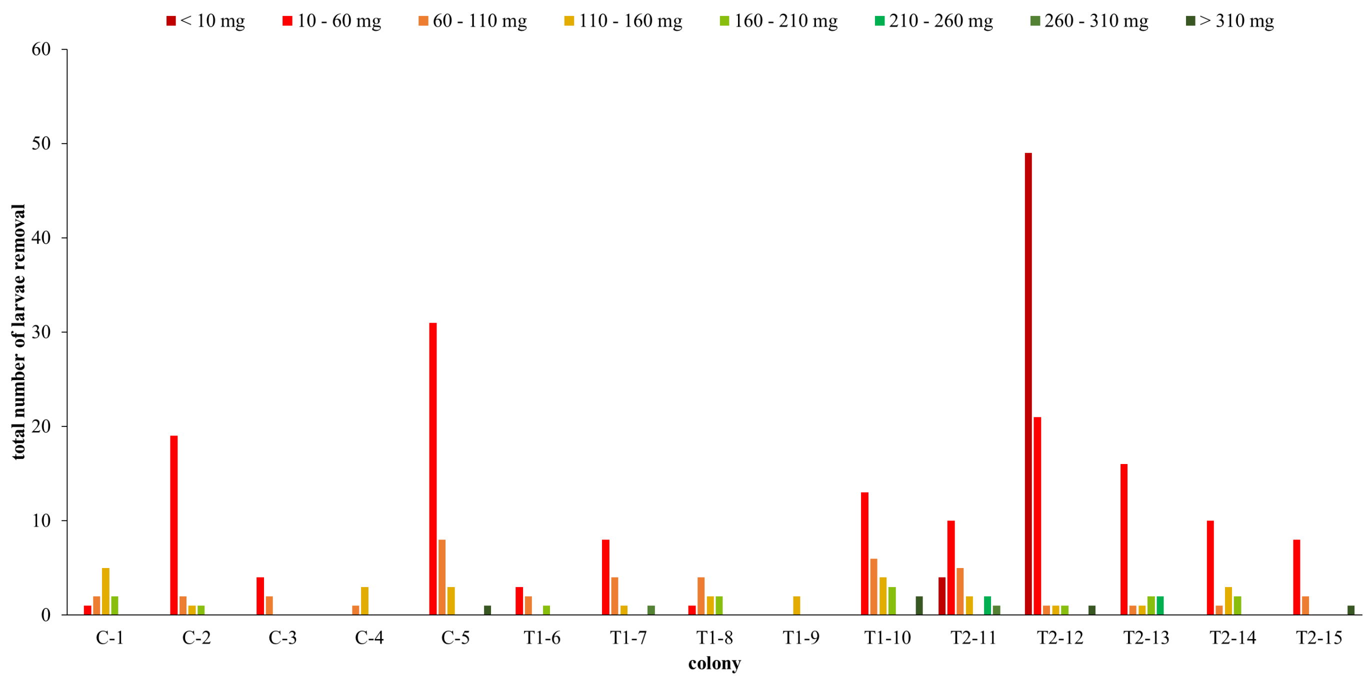

B

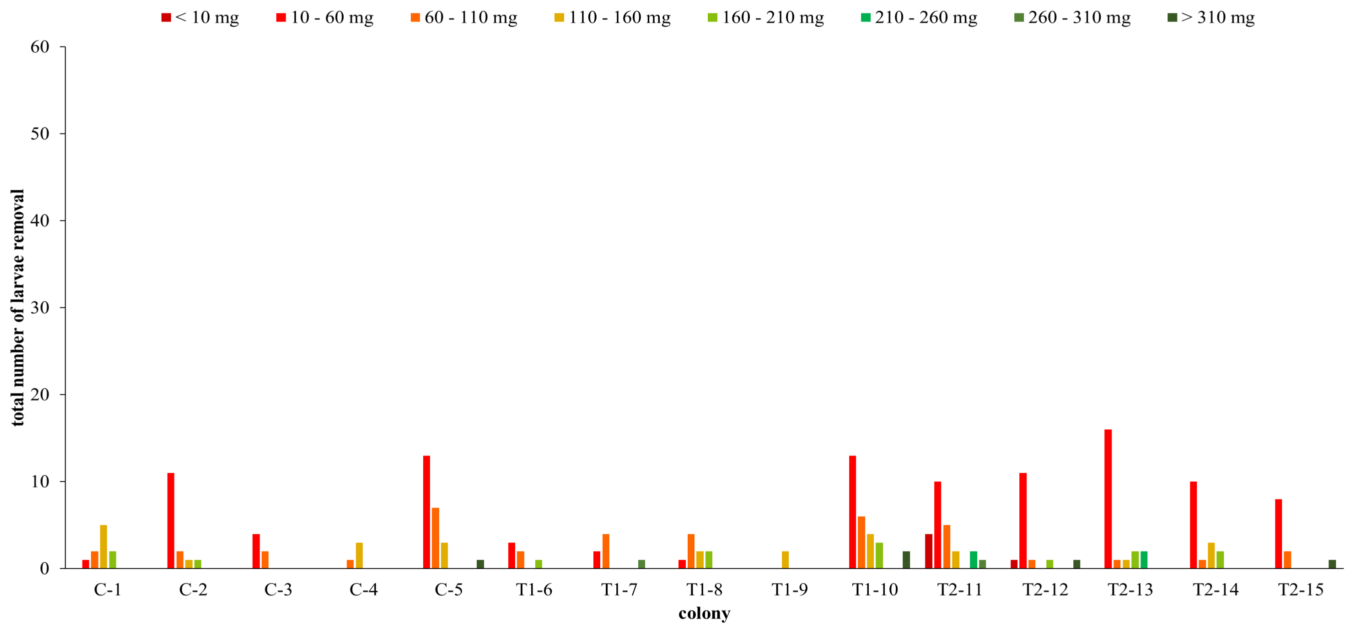

Fig. S4. Larvae removal by larval weight per colony. The number of larvae removed per colony is divided into different size categories. **(A)** with statistical outliers and **(B)** without statistical outliers. (C: control, T1: wounded, T2: exposure to *B. thuringiensis*, 1-15: colony number, see Table 1)

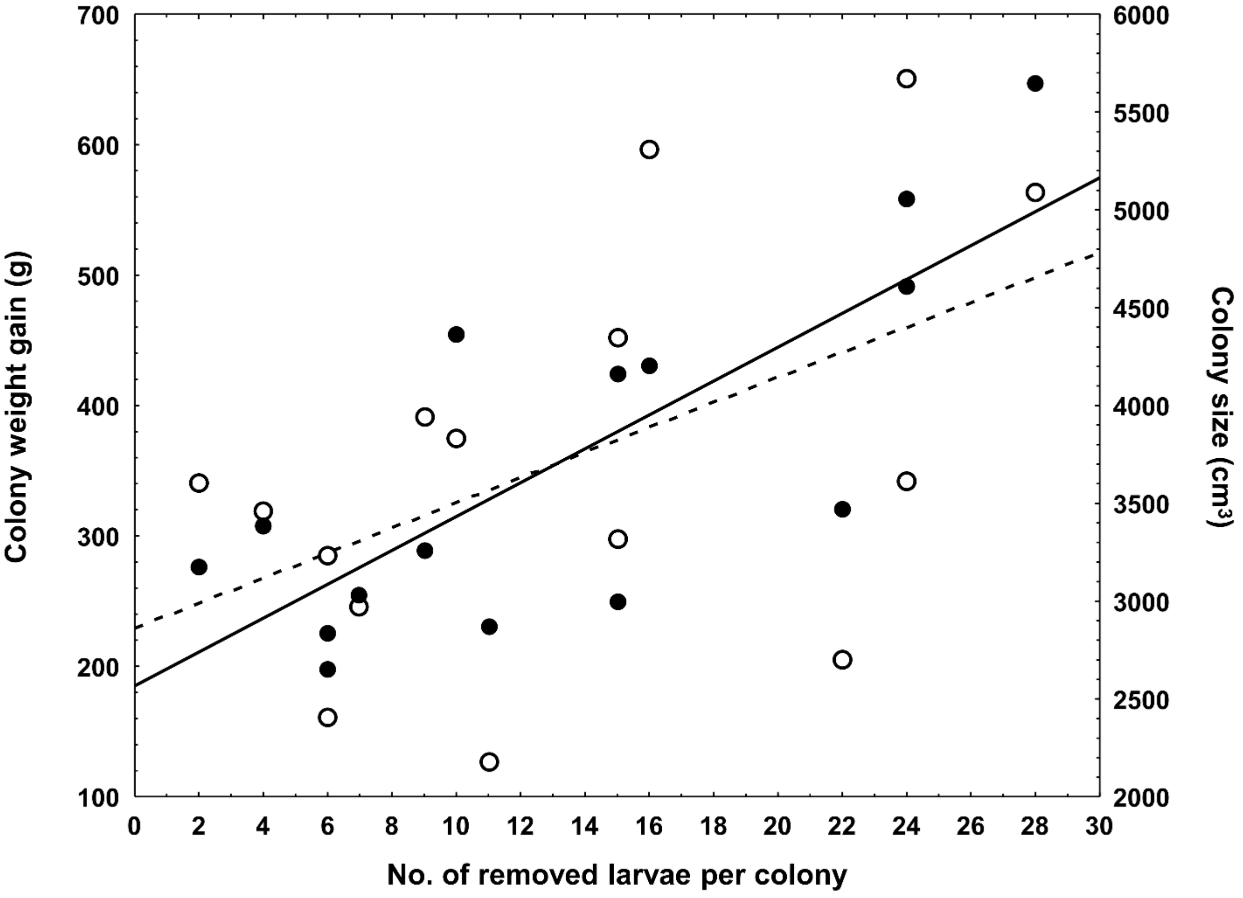

Fig. S5: Relationship between number of removed bumble bee larvae per colony (n = 15 colonies) and colony weight gain (in g, left y-axis, filled circles and solid line, $r^2 = 0.6086$, $p = 0.0006$) as well as colony size on day 15 (in cm^3, right y-axis, empty circles and dashed line, $r^2 = 0.2564$, $p = 0.0541$).

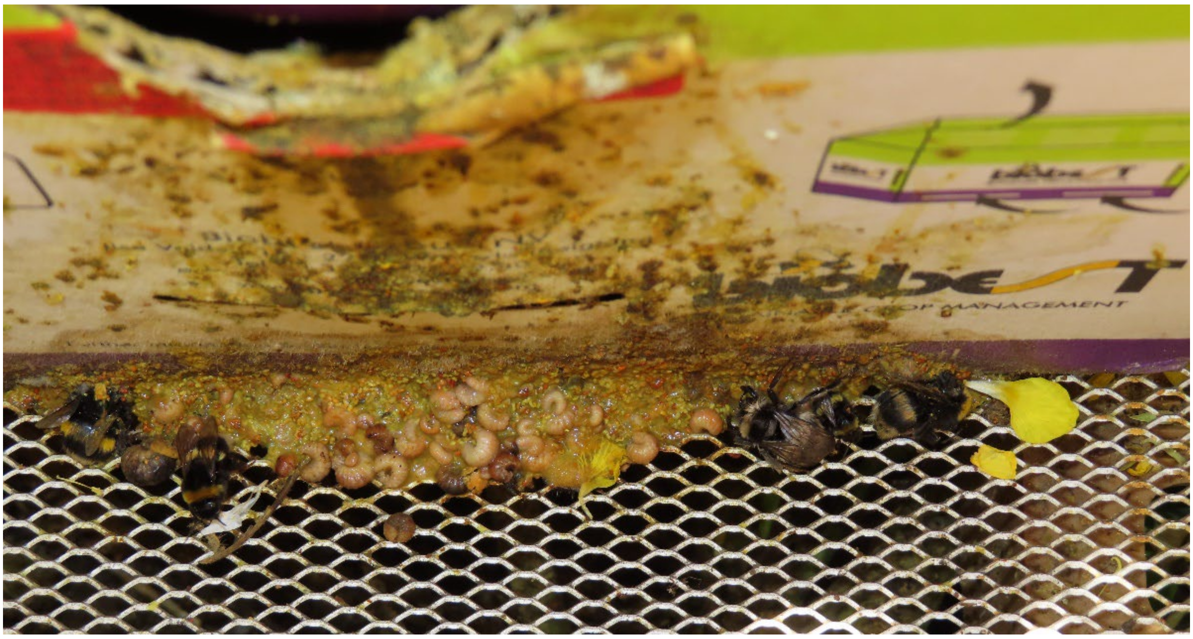

Fig. S6: Differently coloured ejected larvae and of different size outside the bumble bee nesting box. This phenomenon was observed under field conditions where bumble bee workers foraged freely in FlorBac^{TM} treated oilseed rape fields (Alkassab and Wueppenhorst, pers. observation, 2021).

Video S1: Worker bumble bee (*B. terrestris*) removing a large white bumble bee larva from its colony (Wueppenhorst, pers. observation, 2021).

Table S1. PCR results grouped by larval weight and colour for the T2 (bacteria exposure) treatment. **(A)** Number of larvae in the different weight categories tested for *B. thuringiensis* and the number of *B. thuringiensis* positive larvae. **(B)** Number of larvae in the different colour categories tested for *B. thuringiensis* and the number of *B. thuringiensis* positive larvae.

A

Weight category (mg)	No. of tested larvae	No. of *B. thuringiensis* positive larvae
< 10	1	0
10 – 60	21	7
60 – 110	3	2
110 – 160	2	1
160 – 210	2	2
210 – 260	2	0
260 – 310	0	0
> 310	2	0

B

Colour category	No. of tested larvae	No. of *B. thuringiensis* positive larvae
White	16	9
Grey	11	1
Brown	2	0
Black	4	2
